# Supplementary material for: Are carbon emissions trading and green financial instruments synergistic? -Comprehensive quantitative research based on content analysis
Source: PLoS One. 2024 Mar 7;19(3):e0298601. doi: 10.1371/journal.pone.0298601 (PMC10919601; doi:10.1371/journal.pone.0298601)
Supplement: S1 Data — (ZIP) [file pone.0298601.s001.zip › Data/地级市Z_F工作报告.zip!/╜¡╦╒/│ú╓▌╩╨/└┤╫╘╣1⁄2╓┌║┼í╛┬φ┐╦╩2╛▌═°í┐_2006.docx]

各位代表：

　　现在，我代表常州市人民政府，向大会作工作报告，请予审议，并请市政协各位委员和其他列席人员提出意见。

　　一、2005年工作回顾

　　2005年是“十五”最后一年，也是奋发有为的一年。市政府在中共常州市委的正确领导下，在市人大和政协的监督支持下，紧密依靠全市人民，坚持以邓小平理论和“三个代表”重要思想为指导，深入贯彻科学发展观，紧紧围绕“两个率先”，聚精会神抓经济，真抓实干促发展，以民为本办实事，锐意进取，团结奋战，全面完成了市十三届人大四次会议确定的各项目标任务。

　　——国民经济平稳较快增长，人民生活水平稳步提高。预计，全年完成生产总值1302.2亿元，按现行价增长18.1%；人均生产总值超过4500美元；财政总收入达到220亿元，增长25.9%，其中地方一般预算收入95亿元，增长27.4%。农业综合生产能力明显提升，预计实现农业增加值56.5亿元；农业产业化经营步伐进一步加快，形成花卉苗木等优势产业带（区）80多万亩，25家市级以上重点农业龙头企业经营收入突破100亿元；农村集体经济股份合作制改革等“三大改革”稳步推进。工业经济持续增长，预计全部工业总产值突破3000亿元，规模以上工业实现增加值600亿元，按可比价增长23%；新增省级高新技术企业39家、高新技术产品156只，高新技术产业产值占工业产值的比重达32.3%；中天钢铁销售收入达到108亿元，成为我市首家销售超百亿元的企业集团；新增境外上市企业3家、中国驰名商标1只、中国名牌产品8只，入选“中国品牌经济城市”。服务业发展呈现良好势头，增加值达到450.4亿元，增长16.6%；预计全社会消费品零售总额420亿元，增长16.3%；旅游业游客和收入分别增长23%和23.5%。在经济增长的同时，全市人民生活水平得到新的提高，城镇居民人均可支配收入达到14589元，增长13.4%；农民人均纯收入达到6900元左右，增长10%左右；全市城乡居民储蓄余额达到865.4亿元，增长19.5%。

　　——“三加”工程顺利推进，经济结构进一步优化。组织实施以“加快利用外资、加大民资投入、加速园区建设”为主要内容的“三加”工程。全年完成全社会固定资产投资770亿元左右，增长30%以上，其中工业投入450亿元以上，增长32%以上；投资结构明显优化，高新技术产业投入增长三成左右，高能耗、高污染行业投资得到有效控制，全市单位生产总值能耗同比下降2%。利用外资取得重要突破，扭转了年初负增长的不利局面，全年协议利用外资28.5亿美元，增长30%以上，注册外资实际到账8.7亿美元，同比增长50%以上；新批项目单体规模达到570万美元，新批1000万美元以上项目145个，引进总投资超亿美元项目7个，新批服务业外资项目84个，又有5家世界500强企业投资我市。成功举办了中国常州科技•经贸洽谈会和对台招商恳谈会。开发园区建设加快推进，出口加工区获得批准并启动建设，武进海关办事处获准设立。外贸外经稳步增长，完成进出口总额80亿美元以上，增长20%，其中出口超过60亿美元，增长30%左右；实现外经营业额1.9亿美元，增长30%以上，又有10家企业到境外投资。民营经济加速发展，私营企业新增7200户，总数超过4.5万户，注册资本新增110亿元，总额达到580亿元以上；民营经济对全市经济增长的贡献份额进一步提高，实现增加值占全市总量的65%左右。

　　——城市建设步伐加快，城乡统筹协调发展。编制了新的三年城市建设实施纲要，基本完成新一轮城市总体规划中的23个专项规划和镇村布局规划。交通建设加快推进，沪宁高速常州段扩建、312国道和239省道改扩建工程建成通车，录安洲夹江大桥主桥合龙，常宁高速、扬溧高速和新运河建设进展顺利，常州民航机场改造工程开始实施。城市基础设施建设取得新成绩，外环路、青洋路等改扩建工程顺利完成，长江路、常焦路等骨干道路建设按计划推进，常州-金坛供水工程、城市生活垃圾卫生填埋厂二期、江边污水处理厂一期等公用设施建成投运，生活垃圾焚烧发电项目动工兴建，武宜运河武进段拓浚、藻港河南段整治等城市防洪工程全面完成。老住宅小区整治成效明显，全年整治15个老小区120万平方米，改造拆迁危旧房1500户。国家康居示范工程陈渡新苑小区开工建设。城市环境进一步改善，新增城市绿地319公顷，完成荆川公园敞开改造和青枫公园一期工程，建成东方绿地等一批公共绿地和道路景观，启动实施“八路八口”绿化工程、红梅公园敞开扩建等工程。城市管理得到加强，开展了以“限摩”为重点的城市道路交通秩序专项整治，新辟、调整和延伸公交线路17条。村镇建设进一步加快，农村清洁田园、清洁水源、清洁家园的“三清”工程取得初步成效。

　　——社会事业和各项改革全面推进，和谐社会建设成效显著。坚持富民优先原则，全市新增就业6.9万人，城镇登记失业率控制在3.58%；社会保险覆盖面进一步扩大，全市净增参加养老保险8.2万人、医疗保险8万人。提高了城乡低保、农村“五保”和城镇“三无”对象生活补贴标准，建立了以城市低保对象为重点的医疗救助制度，全市93%以上的农民参加了新型农村合作医疗保险。创新慈善劝募活动，慈善创始基金近2亿元，总量名列全省第一。坚持为民办好实事，除因规划调整的河道整治项目外，年初确定的实事项目全部完成。科教兴市战略深入实施，新增市级以上工程技术研究中心6家、企业技术中心18家，获得授权专利达1200件。高质量普及学前三年教育和十二年教育，全市高考万人进线率和单招录取率继续名列全省第一，高等教育毛入学率达到54%；大学城二期建设进展顺利，在常高校学生达到9.5万人，比上年增长22%。全年引进各类人才1.56万名。新闻、文体卫生事业取得新进步，成功举办了第二届中国国际（常州）卡通数码艺术周，20家动画企业入驻常州国家动画产业基地；常州博物馆、前后北岸历史文化街区建设积极推进；市妇幼保健院病房楼、市残疾人康复服务中心建成投运，全市乡镇提前三年达到省农村初保先进地区标准；成功承办全国十运会常州赛区赛事，常州籍运动员取得了6.5枚金牌的好成绩，第九次获得全国全民健身先进城市称号；实施农村部分计划生育家庭奖扶制度，继续保持人口低生育水平。人防、国防和民兵预备役建设得到加强，常州“国防园”建成并投入使用。各项改革取得新进展，国有资产管理体系和公共财政体系进一步健全，企业改革和行政管理体制改革进一步深化，市属生产经营性事业单位改制转企工作基本完成。精神文明和民主法制建设进一步加强，创建成全国文明城市工作先进市，涌现出英雄教师殷雪梅、新时代产业工人代表邓建军等先进人物；积极深化学习型城市建设，公民道德建设和未成年人思想道德建设扎实推进；“法制常州”建设全面启动，圆满完成“四五”普法各项任务；“平安常州”建设取得新成效，大调解机制、社会治安防控体系进一步完善，被评为全国社会治安综合治理优秀市。坚持不懈地抓好安全生产、食品安全、打假治劣和整顿规范市场秩序工作。与此同时，外事、侨务、台湾事务、老龄、地方志、妇女儿童、关心下一代、民族和宗教等工作都取得了新成绩。

　　——政府自身建设得到加强，投资创业环境明显改善。把开展保持共产党员先进性教育活动与加强政府自身建设有机结合起来，强化党的意识、民本意识、法制意识、效率意识、创新意识和廉洁意识，进一步精简会议文件、加快工作节奏、深化政务公开、加强督查落实、树立诚信形象，提升了政府的行政能力和工作水平。按照“法制、诚信、效率、双赢”的要求，着力抓好软环境建设，在“放、减、控、改”等方面取得了突破，把外资审批权等14项市级审批权下放给辖市区；减轻企业负担，常州高新区和武进高新区实行市级行政事业性“零收费”；控制和规范对企业的检查，推行“宁静生产（经营）日”制度；改进工作作风，全面推行企业联合年检、申办告知承诺、建设项目联合审批和收费一卡制等四项制度，对乱收费等行为进行了通报批评。同时，进一步加强廉政建设，自觉接受市人大及其常委会的法律监督和工作监督，支持市政协履行政治协商、民主监督、参政议政的职能，514件市人大、政协议案、建议和提案全部办结。

　　回顾一年来的政府工作，我们的主要体会是“六个坚持”：坚持突出中心抓经济，把更多的精力投入到抓经济、促发展上来，做到一心一意谋发展，专心致志搞建设；坚持扬长避短抓突破，着力解决经济社会发展中的薄弱环节，努力创造自身特色优势，确保常州在区域经济发展中的应有地位；坚持与时俱进抓创新，用新的办法破解发展中出现的各种难题，及时化解经济运行中的矛盾和问题，保持经济社会平稳较快发展；坚持有效推进抓落实，对事关发展全局的重大事项、重大问题，上下同心，全力以赴，狠抓推进，确保各项工作落到实处，取得实效；坚持以人为本抓富民，把发展经济与造福人民统一起来，扎扎实实地为全市人民多办实事好事，让人民群众共享更多的改革发展成果；坚持统筹兼顾抓和谐，积极解决发展中不够协调、不够全面的问题，推动城市与农村、经济与社会、物质文明与精神文明建设协调发展。

　　各位代表，我们所取得的成绩，是全市人民在中共常州市委的正确领导下，团结奋斗、开拓创新的结果，是市人大、政协监督支持的结果。在此，我代表市人民政府，向全市人民，向人大代表、政协委员、各民主党派、工商联、各人民团体、离退休老同志、无党派及各界人士，向驻常部队指战员、武警官兵和部、省驻常单位，向所有关心、支持常州建设和发展的海内外朋友们，表示衷心的感谢！

　　在肯定成绩的同时，我们也清醒地看到工作中存在的问题和不足。主要表现在：一是经济转型面临考验。虽然我市经济一直保持平稳较快的增长势头，但从总体上看，产业层次还不高，特别是制造业竞争能力还不够强，如何走出一条具有常州特色的新型工业化道路，对我们是一个考验。二是薄弱环节依然薄弱。虽然去年利用外资走出了低谷，并有较大幅度增长，但外资规模和质量与先进地区相比仍然存在较大差距。三是富民工程任务艰巨。虽然去年全市城乡居民收入有了新的增长，但增幅还不够高，与今年要全面实现小康指标的要求看，还有一定距离。四是和谐稳定任重道远。就业再就业和社会保障形势不容乐观，水污染和大气污染状况在一些地方还比较严重，环境治理的任务依然艰巨。同时，随着改革的深入，一些历史遗留问题和新的矛盾交织，群访事件时有发生，维护社会稳定的任务十分繁重。五是自身建设亟待加强。对照新的形势和科学发展的新要求，政府组成人员还有很多不适应的地方，转变政府职能、改进工作作风、提高工作效率还有大量的工作要做。这些问题都有待于我们在今后的工作中，采取切实有效的措施，认真加以解决。

　　二、2006年工作安排

　　各位代表，2005年各项任务的全面完成，标志着我市“十五”发展取得圆满结果，“两个率先”迈入新的阶段。“十五”时期是常州发展经历挑战、经受考验最多的时期，也是经济社会发展最快、改革开放成效最好、城乡面貌变化最大、老百姓得到实惠最多的时期之一。面向未来，我们已经站在一个新的历史起点上。根据市委九届十四次全会精神，市政府组织编制了《常州市国民经济和社会发展第十一个五年规划纲要》，现已发给各位代表，提请大会审议。“十一五”是常州加快“两个率先”的关键时期，也是承前启后、为后十年常州经济社会发展打基础、增后劲的重要时期。未来的五年，我市将建成具有较高水平的全面小康社会，为基本实现现代化，建设区域中心城市、现代制造业基地和文化旅游名城奠定坚实基础。

　　蓝图已经绘就，五年看三年，三年看头年。今年是实施“十一五”规划的开局之年，是建设全面小康社会的决战之年，做好今年的经济社会发展工作至关重要。今年政府工作的总体要求是：以邓小平理论和“三个代表”重要思想为指导，认真贯彻党的十六大和十六届五中全会精神，按照市委九届十四次全会提出的要求，坚持以科学发展观统领经济社会发展全局，紧紧围绕“两个率先”、富民强市总目标，推进全面创新，加快有效投入，促进城乡统筹，实现全面小康，推动经济社会全面协调可持续发展，开创我市又快又好发展的新局面。

　　我市经济社会发展的主要预期目标是：

　　——全市生产总值增长13%（可比价）；

　　——地方一般预算收入增长15%以上；

　　——单位生产总值能源消耗降低4%；

　　——全社会固定资产投资增长30%；

　　——社会消费品零售总额增长14%；

　　——进出口总额超过100亿美元，出口总额增长20%以上；

　　——实际到账外资同比增长25%以上；

　　——城镇居民人均可支配收入增长12%，农民人均纯收入增长10%以上；

　　——城镇登记失业率控制在4%左右；

　　——人口自然增长率控制在1‰以内。

　　为实现上述目标，我们将重点抓好以下八项工作：

　　（一）加大有效投入，调整产业结构

　　有效投入是调整经济结构、提升产业层次和壮大新兴产业的重要途径和关键环节。今年我市将深入开展“加快有效投入年”活动，通过有效投入，带动结构调整，促进产业升级。

　　优化投资方向和结构。按照新型工业化要求，根据国家产业政策和我市产业规划，加大对高新技术产业投入，大力扶持电子信息、生物医药和精细化工、新材料等新兴产业，着力推进朗生微电子、天合光能等高新技术重点项目，不断提高高新技术产业投入在全部投入中的比重。加大对传统产业的技改投入，抓好信息产品和信息技术的推广应用，着力推进新型环保制冷剂、数控加工中心等38项重点工业技改项目。加大对循环经济的投入，实施一批节能降耗项目，淘汰一批能耗高、污染重、技术落后的工艺和产品，鼓励企业实施循环式生产，推动产业循环式组合。

　　突出抓好重大项目。项目是有效投入的直接载体。大项目催生大企业，大企业造就大产业。把实施大项目作为有效投入的重中之重，积极推进168个重大项目的实施，力争开工20个总投资超亿美元的项目。进一步加大工业项目的组织力度，重点推进中天钢铁等50个投入规模大、产业层次高、带动能力强的重大项目，加快实施晋美光电等50个发展前景好、产业链长的制造业升级项目。重点实施常州港物流园区等50个现代服务业大项目，确保年内新开工建设22个，投资规模达到112亿元。同时，抓好10个重点城建项目和武南河拓浚等8个重点农林水利建设项目。

　　精心组织“加快有效投入年”活动。按照发动广、规模大、项目多、投向优、成效好的要求，进一步激发全市上下投资创业热情，掀起新一轮加大有效投入的高潮，确保全社会固定资产投资规模突破1000亿元。坚持大中小项目、一二三产业和各种经济成份一起上，通过“创星争先”等激励措施，深入实施民营经济三年跃升计划，促进广大民营企业争创后劲、做优做强，真正成为我市增加有效投入的主力军。全年新增私营企业6000户、个体工商户12000户，新增注册资本60亿元。搞好协调服务，创新考核机制，加强督查落实，不断破解项目推进中遇到的用地、拆迁和融资等难题，为加大有效投入创造良好环境。

　　（二）统筹城乡发展，建设新型农村

　　贯彻工业反哺农业、城市支持农村的方针，以农民增收为重点，进一步加强“三农”工作，实现城乡统筹协调发展。

　　以提高效益为中心，强力推进现代农业发展。优化农业结构和布局，稳定粮食生产，加快经济林果等高效产业带（区）建设，扶持发展规模养殖示范基地和农业科技示范园区，着力推进高效农业规模化。实施一批农业产业化经营项目，做大做强立华畜禽等一批农业龙头企业，扶持发展农产品加工业，提高农产品附加值。依托农业资源特色，以生态和农耕文化为主题，大力发展观光休闲农业，拓展农业增值增效空间。积极开拓国际国内市场，扩大特色农产品出口规模，培育和扶持凌家塘等一批农产品批发市场，拓宽农产品销售渠道。加快农业标准化建设和农产品品牌建设，健全农技农机推广、农产品质量安全和动植物病虫害防控体系，促进农业安全、优质、高效。

　　以城乡一体化为方向，强力推进社会主义新农村建设。按照生产发展、生活宽裕、乡风文明、村容整洁、管理民主的总体要求，进一步完善城乡发展规划和城镇体系规划，加强重点镇、农民居住区布局规划管理，实现城乡规划全覆盖。加快推进农村“三集中”。统筹城乡基础设施和公共事业建设，加大对农村道路、水利、供水、文化、卫生、环保等公共设施投入，努力实现城镇功能互动共享；组织开展对农村河道、厕所、垃圾、道路、家前屋后等环境综合整治，推进生态防护林等六大现代林业工程建设，改善农村生产生活环境和整体面貌。

　　以“多予、少取、放活”为方针，增强农村发展活力。巩固农村税费改革成果，加强农民负担监管和农村财务管理，推进乡镇机构、农村义务教育改革，进一步减轻农民负担。加大农业投入，完善和强化农业补贴政策，加快茅山老区的扶贫开发，扩大公共财政覆盖农村的范围，建立以工促农、以城带乡的长效机制。放开搞活农村二、三产业，积极改善农村金融服务，鼓励农民自主创业。改革劳动力管理制度，加强城乡统一的劳动力市场和农村劳动力资源信息服务体系建设，加大农民就业培训力度，推动农村劳动力向非农产业和城镇有序转移。大力发展各类农民专业合作经济组织，积极探索农村土地股份合作制改革，有条件的村全面完成集体经济股份合作制改革，进一步激活农村经营机制。

　　（三）加强科技创新，促进产业升级

　　加快产业结构优化升级，关键在于科技创新。通过原始创新、集成创新和引进消化吸收再创新，全面增强科技创新能力，提升产业层次和水平。

　　改造传统产业，提升产业层次。坚持以信息化带动工业化，组织实施30个重点技术创新项目，提升纺织、机械、化工等行业的管理信息化、生产自动化、产品数字化、系统集成化水平。强化政策支持，整合科技资源，提高工程机械、输变电和动力机械等装备制造业研发设计、核心器件配套和生产制造的整体水平。全年力争新增省级高新技术企业30家、高新技术产品120只，力争新增中国名牌产品5只、中国驰名商标3只。

　　加强科技创新，增强竞争能力。切实加强企业为主体、市场为导向、产学研相结合的技术创新体系建设，以重点骨干企业为龙头，组织实施一批关键技术、共性技术及重要装备开发项目，进一步提升自主创新能力，推动高新技术产业向自主研发制造转变。促进轨道交通产业技术引进与自主创新相结合，增强轻轨整车研发能力，提高生产制造和系统集成的整体水平；鼓励“三药”产业研发和创制新药，推进成果转化，加快形成产业规模；以软件外包为突破口，提升自主设计和自主创新能力；大力培育数字化音视频、高性能计算机及网络设备等信息产业，加快形成1万台以上的国产网络计算机生产和应用规模。

　　加快技术创新平台建设，提高服务水平。大力推进公共技术创新服务平台建设，新增5个市级以上工程技术研究中心和10个企业技术中心。加强与高校院所的科技对接，大力引进名校名院的研发机构和力量，推进企业与中科院、清华大学等院所的产学研合作，力争建成5个以上中试基地和合作示范企业。以常州大学城和其他在常高校为依托，建设服务全市的公共科技研发和教育培训平台，引进国内外科技创新资源，发展研发经济、总部经济。加大知识产权保护力度，扶持发展创业服务和科技中介组织，加快科技创业孵化基地建设。加大高新技术产业和现代服务业人才的引进和培养力度，着力增强我市自主创新的原动力。

　　（四）突破利用外资，提高开放水平

　　继续把突破利用外资作为经济工作的重点之一，高质量、大规模地引进外资，并以此为龙头带动我市开放型经济健康发展。

　　创新对外招商。把招商引资与招商选资结合起来，实现利用外资规模与质量的双突破。按照我市产业发展方向，依托优势和特色产业，推进产业链招商。重点对欧美、日韩、港台等国家和地区开展专题招商，大力引进世界500强企业和跨国公司的基地型项目。做好对现有外资企业的服务工作，促进外资企业增资扩股、延伸发展。调动民营企业家引进外资的积极性，支持民营企业境外上市和境外招商。加强招商队伍建设，聘请海外招商代理，创新招商机制，确保完成全年实际利用外资11亿美元。

　　加快园区建设。积极实施开发区三年发展纲要，着力提升国家级高新区、省级开发区和乡镇工业集中区的功能规划和产业发展水平。进一步整合各类园区的土地资源，盘活土地存量，科学用好增量，着力提高投入强度和集约开发水平。加快成片土地开发、标准厂房等基础设施建设，不断增强项目承载能力。深入推进沿江开发，加快录安洲开发进程，增强常州港一类口岸功能。高标准建设出口加工区，力争6月底实现封关运作。加快海关、国检武进办事处建设。抓紧常州保税物流中心的申报工作，积极推进“大通关”建设。深化开发园区体制机制创新，进一步提高办事效率。

　　提升外贸外经。坚持以质取胜、科技兴贸，积极应对外贸形势变化，主动适应出口退税率调整，切实转变外贸增长方式，稳定发展一般贸易，扩大提升加工贸易，积极探索技术贸易。着力优化出口结构，进一步提高机电设备和高新技术产品的出口比重。积极开拓国际市场，支持企业境外参展、国际认证和反倾销应诉，形成多元化出口市场格局。支持有条件的企业“走出去”，实现互利互惠和共同发展。同时，进一步巩固和发展国际友城关系，加强对外交流与合作，努力提高常州在国际上的知名度。

　　（五）加快城建步伐，建设现代城市

　　坚持“科学规划、强化功能、注重生态、统筹城乡、创新管理、集约发展”的原则，科学配置城市资源，加大城市建设力度，实现城市建设的新跨越。

　　拉开城市骨架。适应市区面积扩大和现代化特大城市发展的要求，迅速拉开城市骨架特别是大交通骨架，加快形成现代化综合交通体系。着力推进高速公路网建设，建成沪宁高速扩容配套工程，加快建设宁常高速、扬溧高速；加快高等级干线公路建设，推进104国道等5条干线公路常州段改扩建和续建工程；加快常州民航机场改扩建步伐。以打通南北通道为重点，推进城市快速交通系统建设，建成龙江路等5条贯穿南北的主通道，启动实施南北高架一期工程，积极做好城市轻轨的前期工作。

　　突破南北两翼。在增强中心城区功能的同时，大力推进南北两翼建设。武进区重点围绕“三城一中心”，建设以高等职业教育为特色的常州科教城、旅游休闲为主要功能的太湖湾旅游城、宜人居住的西太湖生态城和5.6平方公里的中心区，把武进区建成全市的现代制造业基地，科教、文化的集聚区，具有生态特色的现代化新城区；新北区重点建设国家级高新区和航空港、长江港“一区两港”以及新龙等组团，使新北区成为全市高新技术产业示范区、现代物流集聚区和现代化新城区，从而形成南北两翼与中心城区互相呼应、相得益彰的城市发展新格局。

　　提升城市功能。加快基础设施建设，加速推进运河南移工程，开工建设石化码头二期扩建工程、录安洲通用和石化码头一期工程，建成录安洲夹江大桥；加快完善城市路网，建设太湖路、竹林北路延伸段、勤业路西段、聚湖路、黄河西路等一批城市干道；加快公用设施建设，推进博物馆及规划展览馆、体育中心及会展中心、大剧院和西瀛里地区及前后北岸改造等项目，开工建设天然气利用二期、魏村水厂二期和武进区客运中心，建成新北区客运中心和一批公共停车场。加快中华恐龙园等六大景区建设，推进旅游业与城市建设一体化发展，年接待游客突破1500万人次。加快绿化工程建设，全面完成“八路八口”绿化任务，新增城乡绿地1600公顷；实施“两纵一横”景观道路建设，启动老运河的景观改造，建设马公桥绿地等绿化工程。与此同时，着力推进中心城区污染企业搬迁，努力改善城市水环境和大气质量，抓好西太湖、长荡湖、天目湖的环境保护，积极创建全国生态示范区和国家园林城市。

　　提高管理水平。完善“大城管”机制，强化辖区、街道的基础作用，综合运用法律、行政、市场和数字化等手段，推进城市管理现代化。建立健全社会预警体系和应急救援机制，加快建设城市应急中心。继续开展交通秩序、市容环境综合整治，加大对城郊地带的治理力度，切实改变城郊结合部的环境面貌。深化完善土地收储和经营机制，积极采用股权转让、BOT等方式吸引外资、民资参与建设，加快形成多元化的城市建设投融资机制。深化和完善市场化取向的市政公用事业改革，加快推进市区公交一体化，深化公交行业改革，提高城市资源的运行质量和整体效益。

　　统筹城镇发展。充分发挥常州中心城市的辐射作用，按照现代化中等城市的目标要求，加快金坛和溧阳两市城镇建设，强化规划，拉开框架，增强功能，优化人居环境，提升管理水平。积极开展新型示范小城镇创建工作，集中力量抓好一批在区位、经济、人口、集聚与辐射力上有明显优势的重点中心镇建设，逐步形成区域中心城市、县域中等城市、小城镇与中心村布局合理、相互衔接、功能互补的现代化城镇新格局。

　　（六）贯彻富民优先，共享发展成果

　　坚持以人为本，始终把提高城乡居民生活水平作为政府工作的出发点和落脚点，让全市人民实实在在地享受到改革发展的成果，得到更多的实惠。

　　增加人民收入，提高生活水平。积极推进就业致富，全面落实促进就业的各项扶持政策，强化就业援助和劳动力就业技能培训，加快发展就业容量大的服务业和中小企业，鼓励企业创造更多就业岗位。积极推进创业致富，把创业作为促进城乡居民收入增长的重要途径，大力发展个体私营经济，充分发挥各类要素资本对城乡居民收入增长的重要作用，努力提高资产性、经营性收入占居民收入的比重。积极推进改革致富，继续深化企业改革，建立与企业发展相适应的职工收入增长机制。

　　完善社会保障，关心弱势群体。把建立完善的社会保障体系作为政府的重要职责。进一步扩大社会保险覆盖面，改进养老保险转移办法，逐步做实养老保险个人账户，推进机关事业单位养老保险制度改革，探索建立新型农村养老保险制度；建立城镇居民医疗保障制度，完善新型农村合作医疗制度，逐步提高城乡居民医疗保障水平，将困难、破产、关闭的国有集体企业退休人员全部纳入医疗保险覆盖范围；妥善解决失地农民的基本生活保障问题，把失地农民纳入社会保障体系；健全城乡最低生活保障制度，不断提高城乡低保、城镇“三无”和农村“五保”对象的供养标准和集中供养水平；进一步推进社会救助体系和社会福利体系建设，更好地发挥慈善基金在助医、助学、助残等方面的作用。

　　立足人民得益，为民多办实事。把更多的精力和财力放到提高人民生活水平上，多做让全市人民得益的事情。今年将全力办好与群众利益密切相关的十件实事：（1）努力扩大就业。新增城镇就业4万人，农村劳动力转移就业4万人，援助困难人员就业2000人，下岗失业人员再就业1.5万人。（2）建立规范的城乡救助体系，为困难群众排忧解难。在市、辖市（区）两级现有投入4000万元的基础上，再增加4000万元，用于城乡10万特困及困难群众的救助救济。从今年开始，城乡低保标准每人每月提高20元，年终再增发一个月标准的补贴；对处于低保边缘的市区困难群众年底给予一次性救济；对当年患有癌症、白血病或尿毒症的市区特困群众、困难群众和个人支付医疗费用超过家庭年收入3倍以上的一般群众，年终给予一次性大病补助。（3）提高农村新型合作医疗保险水平，参保率达到95%以上，市、辖市（区）财政的补贴标准在原来的基础上每人每年再提高20元。（4）实施老年关爱工程。启动实施老年大学搬迁；完成33所敬老院的新建、置换改建和扩建改造任务，新增床位2360张，确保“五保”老人集中供养率达到70%以上。（5）改善城市居民居住条件。加快经济适用房和定销房建设，年供应量由原来的30万平方米增加到50万平方米，今年提供经济适用房和廉租房2000套，提供住房公积金个人贷款13亿元。（6）建设“五园一场”，全部免费开放。年内完成红梅公园敞开扩建、新区中心公园敞开改造以及青枫公园二期、蔷薇生态湿地公园、椿桂园和市民广场建设。（7）实施“清水工程”。年内完成市中心区23条河道的专项整治，确保市区主要河道的水环境得到明显改善。（8）加快推进市区公交一体化。年内完成覆盖市区的公交发展规划，调整和延伸公交线路15条，新辟5条，新增和更新公交车150辆，完成43个行政村通公交。（9）切实减轻城乡居民在子女教育上的负担。从今年秋季开始，全市城乡义务教育阶段的学杂费实行免收，由各级政府承担。（10）继续实施“蓝天计划”。确保70%以上的进城务工人员子弟能进公办学校就读；民办进城务工人员子弟学校的生均公用经费，按市定标准由市、区两级财政解决。

　　（七）坚持协调发展，构建和谐常州

　　更加注重经济社会协调发展，大力推进精神文明和民主法制建设，加快和谐社会建设进程。

　　促进社会公平，实现共同富裕。认真解决好人民群众最关心、最直接、最现实的利益问题，妥善处理好社会各方面特别是不同群体的利益关系，确保人民群众的既有利益不受侵害、改革发展的新增利益公平分配、弱势群体的基本利益得到保障。合理调节收入分配，严格执行最低工资保障制度和企业工资集体协商制度，着力提高低收入者收入水平，逐步扩大中等收入者比重，依法调节过高收入。给弱势群体以更多的关爱，确保每个困难家庭有饭吃、有衣穿、有房住，遇到特殊困难有人帮。

　　大力发展社会事业，提高统筹协调发展水平。坚持教育优先发展战略，进一步提高基础教育水平；积极实施教育现代化工程，全面启动数字校园工程；大力发展高等教育和职业教育，进一步完善大学城二期，启动三期工程建设，形成科教集聚优势。深化文化体制改革，大力发展文化影视产业，促进优秀文化产品生产；扩大广电覆盖，活跃基层文化，加强乡镇文化设施建设；推进淹城保护利用步伐，加快“中国历史文化名城”建设。全面构建新型公共卫生体系，加强重大传染病预防控制和农村卫生工作，推进市疾病控制中心等医疗卫生设施建设，完善社区卫生服务体系。积极迎战第16届省运会，全力申办第17届省运会，努力提高竞技体育水平，广泛开展全民健身活动。加强流动人口计划生育管理和服务，继续稳定低生育水平。同时，继续做好外事、侨务、台湾事务、地方志、民族宗教、关心下一代等工作，支持发展妇女儿童、老龄和残疾人事业。

　　加强精神文明和民主法制建设，保持社会和谐稳定。深入开展争创全国文明城市活动，深化学习型城市建设，倡导和弘扬新时期常州市民精神。进一步做好民兵预备役、国防动员和“双拥”工作。坚持依法治市，深入开展“法制常州”建设，全面加强社会治安综合治理，依法严厉打击各类犯罪活动，提升“大防控”水平，保障人民群众安居乐业。加强“信用常州”建设，规范市场经济秩序，营造良好的金融环境。强化食品药品安全监管，高度重视安全生产，防止重特大事故发生。积极构建“大信访”工作机制，依法处理好土地征用、房屋拆迁等涉及群众利益的问题，努力维护社会稳定。大力加强民主建设和社会管理，进一步完善村民自治、社区自治和企业职工代表大会制度。自觉接受市人大的法律监督、工作监督和市政协的民主监督，加强与各民主党派、工商联、无党派人士和人民团体的联系和沟通。高度重视并认真办理人大代表、政协委员的议案、建议和提案。

　　（八）转变政府职能，打造服务型政府

　　政府工作是落实人民代表大会制度的重要载体。做好今年各项工作，实现全年发展目标，必须加强和改进政府工作。

　　创新行政理念。坚持与时俱进，不断强化“五个理念”。一是科学发展的理念，把加快发展和科学发展统一起来，把经济和社会、农村和城市、当前和长远统一起来，努力保持经济又快又好发展；二是以人为本的理念，把人民群众满意不满意、赞成不赞成、拥护不拥护、答应不答应作为政府工作的根本标准，实现好、维护好、发展好最广大人民群众的利益；三是依法行政的理念，坚持法律至上，认真学法、守法、用法，严格按照法律规范办事；四是改革创新的理念，勇于创新，敢于突破，深入推进各项改革，只要对常州科学发展有利、对人民致富有益，就要敢试、敢闯、敢为；五是勤政廉政的理念，在其位，谋其政，尽其责，堂堂正正做人，踏踏实实做事，清清白白做官。

　　改进工作作风。按照“诚信、透明、简化、明确、高效”的要求，加强行政效能建设，不断提高办事效率和工作水平。诚信，就是树立诚信形象，推行服务承诺制，做到一诺千金，凡是承诺了的事情，一定确保做到。能够做到十分，先承诺七分，承诺了七分，争取做到十分。透明，就是扩大政务公开，实行“阳光操作”，不断提高政府工作透明度。简化，就是善于把复杂问题简单化，简化办事程序，提高办事效率。明确，就是推行各级领导责任制和首问负责制，对群众和企业提出的问题，有明确的态度、明确的答复，决不模棱两可，更不敷衍推诿。高效，就是做各项工作都要雷厉风行，按照“马上办”的要求，以最快的速度“办上马”。

　　优化服务环境。政府就是服务，服务就是环境。各级政府机关要强化为基层、企业和人民群众服务的意识。我们的服务要做到“四个一”：第一，满怀一腔热情，企业创造物质财富，人民是我们的衣食父母，我们要带着真切的感情为他们服务，帮他们办事；第二，把握一个重点，企业发展中的难点就是政府服务的重点，我们要急企业所急，想企业所想，千方百计帮助他们化解发展中的难题；第三，坚持一条标准，我们的服务好不好，检验的标准只有一条：企业发展中遇到的困难和问题帮助他们解决了，我们的服务才算到位了；第四，恪守一项原则，就是我们的服务只能无私无偿，任何个人不能回收回报，更不能索取。

　　各位代表，2006年是充满希望、大有作为的一年。我们正站在新的起点，开启新的征程。让我们高举邓小平理论和“三个代表”重要思想伟大旗帜，全面落实科学发展观，在中共常州市委领导下，同心同德，励精图治，开拓创新，扎实工作，为“十一五”发展开好局、起好步，为建设常州美好的今天和灿烂的明天而努力奋斗！
